# Periodontitis and all-cause mortality in 60- and 66-year-old individuals: a 23-year prospective cohort study

**DOI:** 10.2340/aos.v85.46573

**Published:** 2026-07-31

**Authors:** Sladjana Critén, Pia Andersson, Stefan Renvert, Johan Sanmartin Berglund, Bengt Götrick, Viveca Wallin Bengtsson

**Affiliations:** a Faculty of Health Sciences, Kristianstad University, Kristianstad, Sweden; b Department of Health, Blekinge Institute of Technology, Karlskrona, Sweden; c Faculty of Odontology, Malmö University, Malmö, Sweden

**Keywords:** All-cause mortality, epidemiology, older population, periodontitis, polypharmacy

## Abstract

**Objective:**

The present study aimed to investigate the association between baseline moderate-to-severe periodontitis and all-cause mortality over a 23-year follow-up among individuals aged 60 and 66 years.

**Materials and methods:**

This longitudinal study was based on data from the Swedish National Study on Aging and Care in Karlskrona, Blekinge, Sweden. A total of 340 participants aged 60 and 66 years old at baseline (2001–2003) who underwent periodontal and radiographic examination were included. Baseline data on age, sex, number of medications, smoking status, cash margin, and education were collected.

**Results:**

At baseline, 16.2% of participants had moderate-to-severe periodontitis. During the 23-year follow-up, 52.7% of participants with moderate-to-severe periodontitis died. In the adjusted survival analysis, moderate-to-severe periodontitis was associated with an increased risk of all-cause mortality (hazard ratio = 1.56; 95% confidence interval [CI] = 1.00–2.44, *p* = 0.050).

**Conclusions:**

Over 23-year follow-up, baseline moderate-to-severe periodontitis was associated with a higher risk of all-cause mortality among individuals aged 60 and 66 years. However, although the association was statistically significant, any sex-specific findings should be considered exploratory due to the limited number of events and the resulting wide CIs.

## Introduction

Worldwide, the proportion of older people in the population is growing [1]. In Sweden, it is estimated that by 2070, one in four individuals will be over the age of 64 years [2]. As people age, the prevalence of polypharmacy and multimorbidity increases [3]. These conditions pose significant health concerns, particularly among older people [4]. Today, more than 70% of the population aged 65 years and older are experiencing two or more chronic diseases, such as cardiovascular disease, cancer, diabetes, and stroke [5, 6]. These diseases are strongly associated with increased prevalence of polypharmacy and higher mortality risk, posing major challenges for society due to the burden of health costs [3].

Periodontitis is a global health problem affecting between 50 and 82% of the older population [7]. Periodontitis is the predominant cause of tooth loss among individuals aged 65 years and older, leading to masticatory impairments and reduced quality of life [8]. The association between periodontitis and various systemic conditions is well known [9, 10]. In recent years, some evidence also suggests that the risk of mortality may increase among individuals with systemic diseases such as cardiovascular diseases, diabetes mellitus, and respiratory diseases when periodontitis co-occurs [11–15].

Poor periodontal health has been shown to affect both functional ability and mortality among individuals 75 years and older [16]. Furthermore, studies suggest that poor self-rated oral health, resulting from conditions such as complete or partial absence of natural teeth, may be a predictor of mortality risk in individuals aged 60 years and above [17, 18]. In hospitalized older men with COVID-19, the presence of periodontitis was associated with 14.58 times higher odds of death compared to those without periodontitis [19]. Similarly, Zhang et al. [20] reported that individuals 30 years and older with both depression and periodontitis had an increased risk of all-cause and cancer-related mortality. A meta-analysis by Romandini et al. [21] further supports these findings, showing that periodontitis and edentulism are associated with an increased risk of all-cause mortality as well as mortality from cardiovascular diseases and cancer in individuals ≤ 65 years. These findings highlight the importance of addressing periodontitis as a public health concern in efforts to improve oral and general health in older populations. Previous studies have shown that the prevalence of periodontitis increases with age [22–24], which may affect life expectancy. Although the association between periodontitis and mortality has been investigated previously, few studies have combined comprehensive clinical and radiographic periodontal assessment with long-term mortality follow-up in a well-defined cohort of younger older age adults. The present study therefore aimed to examine the association between baseline moderate-to-severe periodontitis and all-cause mortality among individuals aged 60 and 66 years, followed over 23 years.

## Materials and methods

### Study design

This was a longitudinal cohort study based on baseline periodontal data from the Swedish National Study on Aging and Care (SNAC) in Karlskrona, Blekinge, Sweden, and registry-based mortality follow-up over 23 years. It was conducted in accordance with the STROBE guidelines for observational epidemiological studies [25].

### Description of SNAC study population

The SNAC is a longitudinal ongoing population-based study and started in 2001 with the aim of enhancing knowledge about aging in the older Swedish population. The study includes personal interviews, self-administered questionnaires, medical, psychological, and oral health examinations.

The enrollment of the study population was performed during the baseline (2001–2003). Individuals aged 60–96 years (*n* = 2312) were invited via regular mail to participate. These individuals were randomly selected from the Swedish population database within the municipality of the city of Karlskrona. The selection process ensured equal distribution among individuals aged 60, 66, 72, and 78 years (younger cohorts), whereas all individuals aged 81, 84, and 87 years and older were invited to take part. The overall response rate was 62% (*n* = 1402), representing 10% of Karlskrona’s older population. All individuals provided informed consent. Comprehensive information regarding the study population and the data collection methods has been previously reported [26].

### The present study

#### Study sample and rationale for age cohort selection

Participants met the following inclusion criteria: participants who were 60 and 66 years old at baseline (2001–2003), had completed periodontal and radiographic examinations at baseline and had at least two natural teeth. In total, 397 individuals aged 60 and 66 years were available. Of these, 340 (89.7%) met the inclusion criteria and constituted the study population.

The present study was based on data from the larger SNAC cohort but was restricted to the two youngest age cohorts, comprising participants aged 60 and 66 years at baseline. These age groups were selected to create a relatively homogeneous younger-old population and to investigate whether baseline moderate-to-severe periodontitis in early older age was associated with long-term all-cause mortality during a 23-year follow-up. Periodontal status was assessed only at baseline, whereas mortality was ascertained throughout the follow-up period, using registry-based data on date of death.

#### Sample characteristics

In addition to age, data on gender, number of medications, smoking habits, cash margin, and education were collected from the main SNAC questionnaire. The main questionnaire was used as one of the assessment tools for data collection and is completed by the participants as part of the longitudinal SNAC project [26]. Information on date of death was obtained from the regional population register, which is continuously updated.

#### Covariates

Polypharmacy, sociodemographic status and smoking status were included as covariates as they are known health determinants and increase the risk for mortality [27]. Covariate data were collected at baseline only (Table 1).

**Table 1:** Baseline (2001–2003) characteristics among participants analyzed by gender and total (n = 340).

Variables	Men	Women	Total
	*n* = 157 (%)	*n* = 183 (%)	*n* = 340 (%)
Number of teeth, mean (SD)	22.97 (5.65)	22.40 (5.98)	22.66 (5.83)
Range	2–32	2–31	2–32
≥ 5 medications			
Yes	16 (10.2)	37 (20.2)	53 (15.6)
No	141 (89.8)	146 (79.8)	287 (84.4)
Smoking			
Regular	23 (14.7)	21 (11.6)	44 (13.1)
Occasional	8 (5.1)	3 (1.7)	11 (3.3)
Stopped	69 (44.2)	63 (34.8)	132 (39.2)
Never	56 (35.9)	94 (51.9)	150 (44.5)
Can you get within a week 14,000 SEK to cover an unexpected expense?			
Yes	140 (90.9)^c^	149 (83.2)^d^	289 (87.0)
No	14 (9.1)	30 (16.8)	44 (13.0)
Education, *n* (%)			
≤ 9 years	71 (45.5)^a^	109 (59.5)^a^	180 (53.3)^b^
≥ 10 years	85 (54.5)	73 (40.5)	158 (46.7)
Periodontitis			
Yes	32 (20.4)	23 (12.5)	55 (16.2)
No	125 (79.6)	160 (87.5)	285 (83.8)

Periodontitis defined BOP ≥ 10% + PPD ≥ 5 mm at ≥ 2 surfaces + bone loss ≥ 5 mm in at least 10% of surfaces.

In total, a 337 of 340 individuals answered the question about smoking at baseline.

a One missing;

b Two missing;

c Three missing;

d Four missing.

Polypharmacy defined as the daily use of ≥ 5 medications, which is in line with WHO’s assessment of multimorbidity [28], was used as a pragmatic indicator of overall health in the study population. The number of medications was categorized as ‘having five medications or more’, ‘having less than five medications’, and ‘no medication’.

Sociodemographic status included gender, cash margin, and education level. Gender was classified as man or woman. The categorization of cash margin, Can you, within a week, get access to 14,000 SEK for an unexpected expense?’, was ‘yes’ or ‘no.’ Although cash margin is not a clinical measure, it is a widely used socioeconomic indicator that reflects an individual’s financial buffer and material resources, as demonstrated in previous analyses of the cash-margin variable in Statistics Sweden’s Survey of Living Conditions (ULF 2001:2). Education level was based on years of education and categorized ‘education ≤ 9 years’ and ‘education ≥ 10 years’. Smoking statuses were categorized as ‘yes’ (smoking regularly/smoking occasionally) and ‘no’ (stopped smoking/never smoked).

#### Ethics

Ethical approval for the SNAC studies was obtained by the Research Ethics Committee at Lund University, Lund, Sweden (LU 604-00). The Helsinki Declaration [29] was followed, and all participants provided informed consent. The data obtained were coded, and the authors did not have access to personal data (except for age and gender) of the study participants.

#### Periodontal examination

Two experienced dental hygienists conducted the periodontal examination and exposed the panoramic radiographs. The radiographs were analyzed by a periodontist. To enhance reliability for clinical examinations, dental hygienists were calibrated prior to the study start at baseline 2001–2003. Concerning periodontal probing depth (PPD), an intraclass correlation coefficient (ICC) was calculated based on measurements from 14 participants. The ICC was 0.76 (95% CI = 0.67–0.82; *p* < 0.001). The clinical variables and outcome measures in the present study included: number of teeth, bleeding on probing (BOP), PPD ≥ 5 mm, and level of alveolar bone ≥ 5 mm from cement-enamel junction (CEJ) to the highest marginal bone level mesial and distal on each tooth. Full mouth measurements of BOP and PPD were performed on four surfaces per tooth (mesial, buccal, distal, and lingual) using a periodontal probe CP-12 (Hu-Friedy Inc., Chicago, IL). The proportion of BOP at the site with the worst bleeding was registered. The deepest PPD ≥ 4 mm on each tooth was registered. Only PPD ≥ 5 mm at the tooth level was included in the analysis.

The radiographic assessments were conducted by an independent periodontist who was blinded to all personal and medical information for all performed readings. On the panoramic radiographs, the alveolar bone level was measured using a transparent millimeter-graded ruler, 2x magnification, and a light box source. The measurements from the CEJ to the highest marginal bone level, both mesially and distally on each tooth, were recorded. To minimize the influence of radiographic distortion, magnification, and an estimated measurement error of ±1 mm, we considered that a distance from the CEJ to the most coronal interproximal bone level of 5 mm indicated bone loss [30]. A reliability analysis was performed on a random sample of cases with repeated radiographic evaluations. The ICC for alveolar bone level ≥ 5 mm from the CEJ to the highest marginal bone level was 0.97 (95% confidence interval [CI]: 0.92–0.99; *p* < 0.001).

The definition of periodontitis used in this study was adapted from the American Academy of Periodontology (AAP)/European Federation of Periodontology (EFP) classification [31]. Because clinical attachment level (CAL) measurement was unavailable, moderate-to-severe periodontitis was defined as BOP ≥10% in combination with PPD ≥5 mm and radiographic alveolar bone loss ≥5 mm in at least 10% of tooth surfaces. Consequently, the exposure variable in the present study should be interpreted as moderate-to-severe periodontitis rather than mild or early-stage periodontitis. The inclusion of BOP ≥10% ensured the presence of gingival inflammation, while the requirement of ≥ 10% of surfaces with ≥ 5 mm bone loss ensured that periodontal destruction was present on at least two non-adjacent teeth. This criterion was applied to reduce the risk of classifying localized deep pockets on adjacent teeth as generalized disease. For the purpose of the present analyses, periodontitis was treated as a dichotomous baseline exposure (moderate-to-severe periodontitis: yes/no), and no further categorization according to disease severity or stage was performed.

### Statistical analysis

Descriptive and analytical statistics were performed using the Statistical Package for the Social Sciences (IBM SPSS, version 27.0). Frequencies and percentages were used to summarize age groups and data based on baseline and 23-year follow-up in relation to periodontitis. The proportional hazards assumption was assessed first visually for all covariates using log-minus-log survival plots in a Cox regression model and second using scaled Schoenfeld residuals (Grambsch-Therneau test) implemented via the cox.zph function in the survival package in R (version 4.5.0). A two-sided *p*-value below 0.05 was considered indicative of assumption violation.

In survival analyses, the Cox regression model based on hazard ratio (HR), CIs, and *p*-values [32] was used to calculate if there were associations between a primary covariate (gender, ≥ 5 medications, cash margin, education, smoking, and moderate-to-severe periodontitis), and time of death at the study endpoint. Dichotomous dependent variables were coded as 0 (alive) and 1 (death). The analyses of time events included the study start or the date for periodontal examination. Person-days from the study start to the endpoint or the earliest event, either the date of death or the date of the last follow-up, were calculated. Individuals aged 60 and 66 years were analyzed as one group. The primary outcome was all-cause mortality. Because all deaths were counted as events, competing risks were not modeled in the primary analysis. Cause of death was unavailable, which precluded cause-specific competing risk analyses. In additional exploratory analyses, periodontal disease burden was assessed using continuous periodontal variables. The number of teeth with PPD ≥ 5 mm and the number of sites with radiographic bone loss ≥ 5 mm from the CEJ were analyzed in separate Cox regression models. These variables were analyzed separately because they reflect related aspects of periodontal disease burden. To investigate whether gender modified the association between moderate-to-severe periodontitis and all-cause mortality, an interaction term between gender and moderate-to-severe periodontitis was included in the Cox regression model. An interaction term between gender and baseline periodontitis was calculated and included in the model together with the main effects of gender and periodontitis. Gender was coded as women = 0 and men = 1 and moderate-to-severe periodontitis as no = 0 and yes = 1. These models were adjusted for the same covariates as in the fully adjusted model. Statistical significance was determined at *p* < 0.05.

Missing data were handled using complete case analysis in the Cox regression models. Participants with missing information on baseline periodontitis or any of the covariates included in the model were excluded from the analysis. Participants who did not die during follow-up were censored at the end of the 23-year observation period.

## Results

### Baseline characteristics

In total, periodontal and radiographic records of 340 individuals (53.8% women) were included. Of these, 165 were aged 60 years and 175 individuals were 66 years. Of these individuals, 262 (77.1%) had ≥ 20 teeth (121 men and 141 women) (not presented in tables). Baseline characteristics of study individuals, in relation to age and gender, are presented in Table 1.

### Prevalence of moderate-to-severe periodontitis

In the entire population, periodontitis was present in 55 (16.2%) individuals (see Table 1). From these, 19 (34.5%) smoked regularly, 7 (12.7%) smoked occasionally, 15 (27.3%) had stopped smoking, and 14 (25.5%) never smoked. Forty-two individuals (76.4%) had ≤ 9 years of education, 9.1% used ≥ 5 medications per day, and 12 (22.2%) individuals were not able to cover 14,000 Swedish crowns in unexpected expenses within a week.

### Association with all-cause mortality

No significant violations of the proportional hazards assumption were detected – moderate-to-severe periodontitis: chi² = 1.45, *p* = 0.229; polypharmacy: chi² = 0.00, *p* = 0.987; cash margin: chi² = 0.10, *p* = 0.758; smoking: chi² = 1.70, *p* = 0.192; education: chi² = 3.73, *p* = 0.054; gender: chi² = 1.12, *p* = 0.290; and global test: chi² = 8.35, df = 6, *p* = 0.214).

Cox regression analysis at baseline was used for moderate-to-severe periodontitis as an independent variable and time for the first event (death) as the dependent variable with adjusted covariates: gender, number of medications, cash margin, education level, and smoking (see Table 2). In the final Cox regression model, 330 of the 340 baseline participants were included in the analysis, while 10 participants (2.9%) were excluded due to missing values. During the 23-year follow-up, 135 deaths (66 men and 69 women) occurred, and 195 participants were censored (86 men and 109 women) (not presented in table or figure). Overall, of those who had moderate-to-severe periodontitis at baseline, 29 of 55 (52.7%, 14 women and 15 men) died, and of these 13.8% had polypharmacy (four women) (not presented in table or figure).

**Table 2:** Associations between a given event (death) and moderate-to-severe periodontitis as an independent variable, including gender, use of five medications or more, cash margin, education, and smoking (*n* = 340).

Independent variables	All individuals			Men			Women		
	*n* = 340^a^			*n* = 157^a^			*n* = 183^a^		
	HR	95% CI	*p*	HR	95% CI	*p*	HR	95% CI	*p*
Periodontitis	1.56	1.00–2.44	**0.050**	1.04	0.54–2.01	0.906	2.45	1.28–4.68	**0.007**
Gender	1.29	0.90–1.84	0.169	-	-	-	-	-	-
Use of five medications or more	1.70	1.10–2.63	**0.016**	2.17	1.06–4.46	**0.035**	1.61	0.94–2.78	0.084
Cash margin	0.68	0.42–1.11	0.124	0.38	0.18–0.83	**0.015**	0.89	0.46–1.71	0.723
Education	1.01	0.91–1.11	0.858	1.03	0.89–1.19	0.716	0.95	0.82–1.09	0.455
Smoking	0.96	0.80–1.16	0.697	0.94	0.72–1.22	0.637	1.04	0.79–1.37	0.777

For survival analysis, Cox regression model was used. HR: hazard ratio; 95% CI: 95% confidence interval; BOP: bleeding on probing; PPD: periodontal probing depth. Survival analysis is calculated in days until the first event.

Periodontitis defined BOP ≥ 10% + PPD ≥ 5 mm + bone loss ≥ 5 mm in at least 10% of surfaces.

Significance level *p* < 0.05 presented in bold.

a Missing values (5 men and 5 women): In total, 330 of the 340 participants at baseline were included in the final Cox regression model.

**Figure 1:**
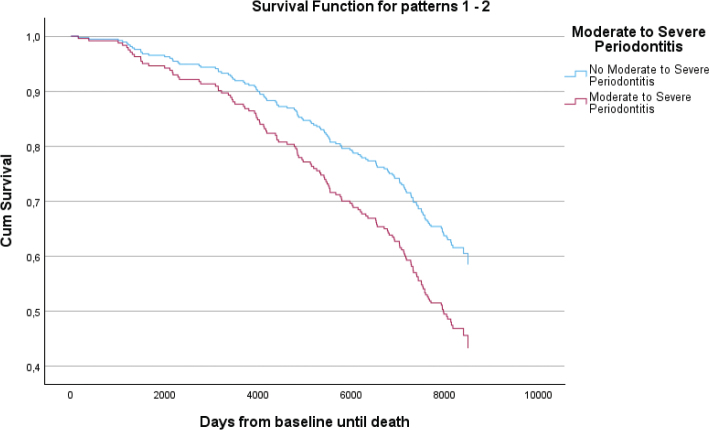
Cox regression curves: 23-year cumulative death survival in individuals with and without moderate-to-severe periodontitis analyzed in total.

**Figure 2:**
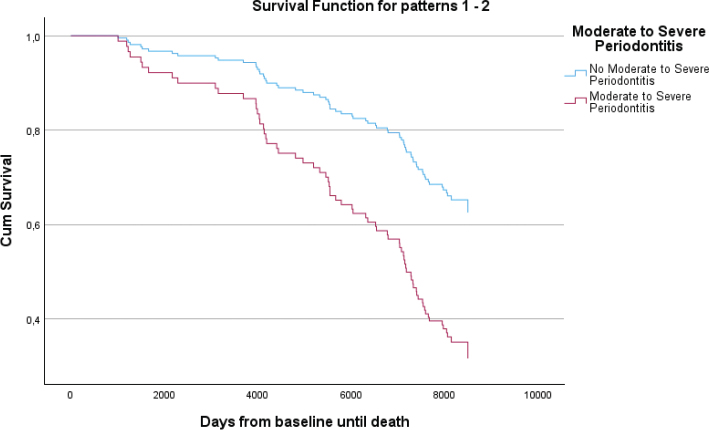
Cox regression curves: 23-year cumulative death survival in women with and without moderate-to-severe periodontitis.

In the fully adjusted Cox regression model including gender, use of ≥5 medications, cash margin, education, and smoking, baseline moderate-to-severe periodontitis was associated with all-cause mortality at the threshold of statistical significance in overall population (HR = 1.56 [95% CI = 1.00–2.44, *p* = 0.050]) and among women (HR=2.45 [95% CI = 1.28-4.68; *p* = 0.007]) (see Table 2; Figures 1 and 2). In additional exploratory Cox regression analyses, periodontal disease burden was further examined using continuous variables: ‘the number of teeth with PPD ≥5 mm’ and ‘the number of sites with radiographic bone loss ≥5 mm’. Each additional tooth with PPD ≥5 mm was associated with an increased mortality risk, HR: 1.09 (95% CI: 1.03–1.15; *p* = 0.001), after adjustment for gender, polypharmacy, cash margin, education, and smoking. Similarly, the number of tooth sites with radiographic bone loss ≥5 mm from the CEJ was significantly associated with all-cause mortality. Each additional tooth site with radiographic bone loss ≥5 mm was associated with an increased mortality risk, HR: 1.06 (95% CI: 1.03–1.10; *p* < 0.001), after adjustment for gender, polypharmacy, cash margin, education, and smoking. However, the gender and periodontitis interaction was not statistically significant, HR = 0.51 (95% CI = 0.22–1.19; *p* = 0.118), indicating no clear statistical evidence that the association differed between men and women.

## Discussion

The results from this study with 23-year mortality follow-up showed a borderline association between baseline moderate-to-severe periodontitis and an increased risk of all-cause mortality overall and among women. The association persisted even after adjustments for several potential covariates, such as gender, use of ≥ 5 medications, cash margin (14,000 Swedish crowns within a week), education, and smoking, which indicates that periodontitis may have a significant implication on survival in the study population.

The association between moderate-to-severe periodontitis and all-cause mortality among an older population in the present study is in line with other population-based studies [33–37]. Although the results align with previous research, there are still some discrepancies that are worth discussing. Most of the studies mentioned above have investigated the association between periodontitis and mortality in one-way relationships, often in the context to other comorbid conditions such as cancer [34, 36], cardiovascular diseases [33, 35, 36], and diabetes mellitus [37]. In contrast to these studies, the present study has not investigated the cause of death. Furthermore, we focused solely on baseline moderate-to-severe periodontitis as an independent risk factor for all-cause mortality without placing it within the context of other health conditions.

In this study, women with moderate-to-severe periodontitis had a two-fold increased risk of all-cause mortality over 23 years in comparison to men, which is an opposite finding to other population-based studies. Bengtsson Wallin et al. [14] investigated the risk of mortality in individuals ≥ 60 years with periodontitis over 17 years. They found that men had an increased risk of death (HR: 1.5, CI: 1.1–1.9, *p* < 0.006) in comparison to women. In another 7-year follow-up study by Chung and Chen [38], men ≥ 65 years with periodontitis had an increased risk of death (HR: 1.696, CI: 1.606–1.791, *p* < 0.05) in comparison to women. However, a possible explanation for the differences between our results and those from previous studies may be related to the choice of definitions of periodontitis. In the present study, the most recent classification of moderate-to-severe periodontitis, based on BOP ≥ 10% + PPD ≥ 5 mm + bone loss ≥ 5 mm in at least 10% of the surfaces, was used. In contrast, the aforementioned studies applied other classifications. For the definition of periodontitis, Bengtsson Wallin et al. [14] used panoramic radiographic measurements of alveolar bone loss ≥ 5 mm from the CEJ to the marginal bone level at ≥ 30% of sites. Additionally, in the study by Chung and Chen [38], the definition of periodontitis was based on classification from the patient records as ‘abnormal periodontal status’ ‘tooth mobility or periodontitis’. Furthermore, these studies focused on more heterogeneous and larger age groups than the present study, which investigated a smaller number and narrower age range of older individuals with around 9% more women than men. These differences in the definition of periodontitis and sample composition may have contributed to the observed sex-specific associations with all-cause mortality between the present study and studies by Bengtsson Wallin et al. [14] and Chung and Chen [38].

In the present study, analysis revealed that one-sixth of the study population (15.6%) used five or more prescribed medications. In contrast to our result, community-dwelling populations aged 65–74 years, 75–84 years, and 85+ years across 27 European countries had a higher prevalence of polypharmacy (ranging between 25.0 and 51.8%) [39]. The reason for discrepancies between our study and that one may be attributed to several factors such as variances in age, health conditions, and regions where the studies are conducted but most probably due to a younger age cohort. The present study investigated a younger old age group from a limited geographical area in comparison to previous multinational studies with broader age groups and more diverse populations. Additionally, Bonano et al. [39] also studied the prevalence of polypharmacy in relation to several major factors including sociodemographic, mental and physical health, behavioral factors, and physical and living conditions. Furthermore, in our study, the choice of polypharmacy was used only as a proxy for multimorbidity. This approach enabled us to account for general health conditions in the participants at baseline rather than focusing solely on age and gender, which may also explain the differences between the present study and the study by Bonano et al. [39].

It is relevant to highlight that periodontitis is one of the most common oral diseases initiated by subgingival microbiota from dental plaque and the host response [8]. Importantly, periodontitis is a complex condition that triggers the release of various proinflammatory mediators, which not only negatively affect periodontal outcomes [40] but are also closely linked to mortality [41]. In the present study, more than half of the population with baseline periodontitis died within 23 years, and 13.8% of them had polypharmacy. It should be emphasized that factors such as general health conditions, lifestyle, and oral hygiene are shared risk factors for both oral and general health [42]. Therefore, preventing periodontitis has a value in promoting both oral and general health in the older populations and may contribute to reducing health-related mortality risks. Furthermore, gender differences in oral and general health outcomes are important modifiers, and to increase knowledge in this area, attention to gender in future research on periodontitis and mortality is needed. According to Crimmins et al. [43], examining differences in health outcomes between men and women over time, in combination with historical data on life expectancy, disease, and behavioral conditions, as well as recent data on biomarkers, would give a clearer understanding of when and where these differences occurred. This would also be significant for understanding the link between periodontitis and mortality.

### Strengths and limitations

The main strength of this study is its longitudinal approach. Studying the narrower age range of younger older individuals over prolonged periods of 23 years provided insight into the association between baseline moderate-to-severe periodontitis and long-term mortality. The additional strength was that periodontal assessment was based on a full-mouth examination, including radiographic assessments, which minimizes the risk for bias and strengthens internal validity.

There are limitations in the present study that need to be highlighted. Missing data were minimal in the final Cox model, as only 10 of 340 participants (2.9%) were excluded due to missing values. Although the impact on the estimates is likely limited, complete case analysis may still introduce some bias if participants with missing data differed systematically from those included. The adapted case definition used in this study most likely captured moderate-to-severe periodontitis rather than mild or early-stage periodontitis, and the inclusion criteria are based solely on baseline data. Another limitation of the study is that cause of death was not available, and therefore competing risks in the sense of cause-specific mortality could not be assessed. The analyses were restricted to all-cause mortality, which provides an overall survival estimate but does not separate potential disease-specific pathways. Additionally, we have no information on whether participants with moderate-to-severe periodontitis at baseline underwent any periodontal treatment during the 23-year observation period, which could influence periodontitis progression. Regarding smoking, we only had information on current vs. nonsmoking status, without any details on smoking intensity, duration, or cessation, which could affect both moderate-to-severe periodontitis and mortality.

Polypharmacy was included as a pragmatic indicator of overall health burden and multimorbidity in this older cohort. While this approach is justified in analysis of all-cause mortality, it is not equivalent to adjustment for specific comorbidities including cardiovascular disease, diabetes, or cancer. Therefore, residual confounding by underlying disease burden remains possible. Lastly, baseline moderate-to-severe periodontitis was present in a small number of participants, and the interaction between men and women was not statistically significant; the observed differences in survival should therefore be interpreted with caution.

Despite these limitations, our findings emphasize the importance of considering periodontal health in older populations, as poor periodontal health is associated with increased long-term mortality risk. In future longitudinal studies, both periodontitis and polypharmacy should be examined over time, as both tend to increase with advancing age. Furthermore, as this is an epidemiological study, causality cannot be established. To clarify the causal relationship between periodontitis and mortality and to explore potential sex differences, long-term cohort studies with repeated periodontal (using stages and grades) and general health assessments are warranted.

In conclusion, over 23 years of follow-up, baseline moderate-to-severe periodontitis was associated with a higher risk of all-cause mortality among individuals aged 60 and 66 years. However, although the association was statistically significant, any sex-specific findings should be considered exploratory due to the limited number of events and the resulting wide CIs.

## Supplementary Material



## Data Availability

All data relevant to the present study are included in the manuscript. The original data are from the SNAC-B project, a population-based study. Researchers need to obtain approval from the SNAC-B data management and maintenance committee in order to gain access to the data. To apply for access, an application for access can be submitted to Ulrika Isaksson (ulrika.isaksson@bth.se) at Blekinge Institute of Technology.
