# Supplementary material for: Periodontitis and all-cause mortality in 60- and 66-year-old individuals: a 23-year prospective cohort study
Source: Acta Odontol Scand. 2026 Jul 31;85:46573. doi: 10.2340/aos.v85.46573 (PMC13434928; doi:10.2340/aos.v85.46573)
Supplement: Supplementary file 1 [file AOS-85-46573-s1.pdf]

Supplementary material has been published as submitted. It has not been copyedited or typeset by Acta Odontologica Scandinavica.

Supplementary analysis to study "Periodontitis and All-Cause Mortality in 60- and 66-Year-Old Individuals: A 23-year Prospective Cohort Study"

Statistical findings by RMST analyses

RMST analyses were performed at 10-, 15-, and 20-year horizons for the total sample and stratified by sex (survRM2 package, R 4.5.0). Maximum observed follow-up was 23.7 years, making all three horizons feasible. Results are presented in the table below. Bold rows indicate $p < 0.05$. RMST analyses were performed at 10-, 15-, and 20-year time horizons in the total sample and stratified by sex using the survRM2 package in R (version 4.5.0). The maximum observed follow-up was 23.7 years, allowing estimation at all three horizons. Results are presented in the table below.

Group	Horizon	RMST moderate to severe periodontitis	RMST no moderate to severe periodontitis	Difference (days)	95% CI	p
Total	10 years	3,351	3,547	-196	-393 to 1	0.051
	**15 years**	**4,726**	**5,157**	**-432**	**-810 to -53**	**0.026**
	**20 years**	**5,928**	**6,556**	**-628**	**-1,206 to - 51**	**0.033**
Men	10 years	3,463	3,491	-28	-243 to 186	0.796
	15 years	4,924	5,029	-104	-547 to 338	0.644
	20 years	6,171	6,331	-160	-858 to 539	0.654
**Women**	**10 years**	**3,195**	**3,590**	**-395**	**-759 to -32**	**0.033**
	**15 years**	**4,449**	**5,258**	**-809**	**-1,471 to - 146**	**0.017**
	**20 years**	**5,589**	**6,732**	**-1,143**	**-2,130 to - 156**	**0.023**

RMST = restricted mean survival time (days).

*Difference = RMST(moderate to severe periodontitis) minus RMST(no moderate to severe periodontitis). CI = confidence interval. Statistically significant results $p < 0.05$ are shown in **bold.***

In the total sample, the 15- and 20-year RMST differences reach statistical significance (-432 days, p = 0.026; -628 days, p = 0.033). The 10-year difference falls short of significance (-196 days, p = 0.051).

Sex-stratified RMST analyses showed significant differences at all three horizons among women, whereas no significant differences were observed among men. However, the sex interaction × moderate to severe periodontitis in the Cox model was not statistically significant (p = 0.118), suggesting limited power to detect interaction. The sex-stratified RMST findings should therefore be interpreted descriptively.
